# Spinal Adhesive Arachnoiditis Mimicking a Spinal Tumor: A Case Report

**DOI:** 10.7759/cureus.87638

**Published:** 2025-07-09

**Authors:** Jotdeep S Bamrah, Vikrant Keshri, Deepak Joshi, Sarav Bamania

**Affiliations:** 1 Neurosurgery, Smt. B. K. Shah (SBKS) Medical Institute and Research Centre, Sumandeep Vidyapeeth (Deemed University), Vadodara, IND

**Keywords:** adhesiolysis, duroplasty, pain, paraparesis, shunt, spinal adhesive arachnoiditis

## Abstract

Spinal adhesive arachnoiditis (SAA) is a rare condition of the meningeal layers of the spinal cord and nerve roots, which can lead to disability. Our patient was a 44-year-old female with a history of cervical spine surgery, followed by external lumbar drain insertion for cerebrospinal fluid (CSF) diversion three years ago. She presented with gradually worsening back pain and bilateral leg weakness. Initial evaluation and MRI of the lumbosacral spine suggested the presence of a spinal tumor. She underwent lumbar spine surgery. But no tumor was identified intra-op; instead, dense arachnoid adhesions encasing the nerve roots were identified, for which partial adhesiolysis was done. Surprisingly, the retained proximal catheter of the lumbar drain was found embedded within adhesions encasing the nerve roots. How it was left in place and missed during the previous drain removal remains unknown and is not documented in the prior treatment records. Though MRI is the most sensitive and specific diagnostic imaging, SAA can mimic an intradural tumor. Despite current knowledge of the nature of adhesive arachnoiditis, as well as advances in treatment and intensive rehabilitation, there are still no established guidelines for prophylaxis, and neurological improvement remains rare. This case report highlights the possible pathophysiology, causes of SAA, and treatment options available for this rare condition.

## Introduction

Spinal adhesive arachnoiditis (SAA) is a rare inflammatory condition of the meninges. It was described as “meningitis serosa spinalis” by Adler and Mendel, and as “chronic spinal meningitis” by Sir Victor Horsley [1], causing inflammation of the pia-arachnoid membrane of the spinal cord and nerve roots, with cystic changes in the subarachnoid space, leading to a thick arachnoid scar tissue. This causes cerebrospinal fluid (CSF) flow abnormalities, which can produce high-pressure pulsations in the subarachnoid space, which can force fluid entry into the spinal cord, causing cord edema and cystic changes [2]. As described in the available literature, the symptomatology varies significantly in terms of presentation, with a delay between the possible initial insult and the development of neurological manifestations ranging from a week to months to years-from asymptomatic, through clinical symptoms with back pain, urinary urgency or incontinence, paresthesias in limbs, hypoesthesia below the affected levels, radicular pain, to severe disability caused by paraparesis/plegia [3,4]. An important risk factor is iatrogenic insult caused by interventions, such as injections of lipid-based contrast agents, lumbar drain insertion for CSF diversion, or epidural anesthesia [4]. Diagnosis is confirmed by assessment of clinical presentation and magnetic resonance imaging (MRI) of the spine showing contrast-enhanced thickened meninges [3,4].

## Case presentation

A 44-year-old female with a short neck, progressive difficulty in walking, with gait instability was diagnosed and treated as a case of craniovertebral junction anomaly three years ago at a different center. MRI during the initial workup was suggestive of atlantoaxial subluxation with atlanto-occipital assimilation, platybasia, basilar invagination, and tonsillar herniation into the cervical spinal canal. She underwent occipito-C2 fixation with insertion of a 3.5 mm spacer on the left side, C1 lateral mass resection, and transoral odontoidectomy, along with fascia lata graft repair of the dura. An external lumbar drain was inserted temporarily for CSF diversion to prevent CSF leak from the dural repair site during post-op healing.

**Figure 1:**
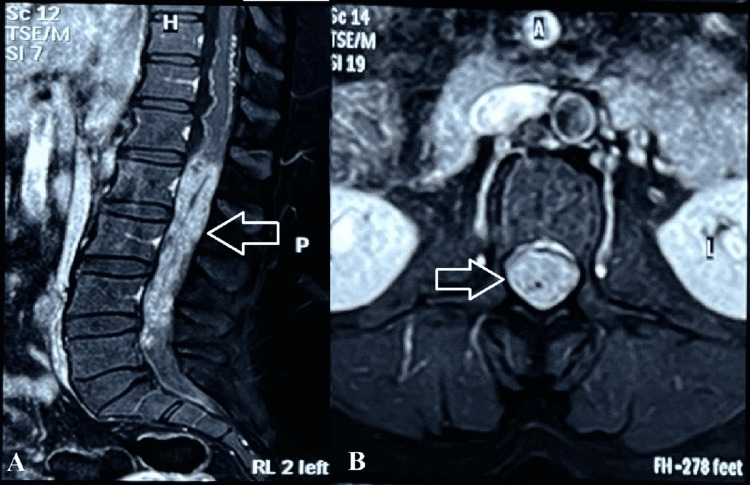
Post-gadolinium contrast T1-weighted MRI sequences show intradural enhancement extending from the L1 to L5 levels in the sagittal (A) and at the L2 level in the axial (B) cuts.

**Figure 2:**
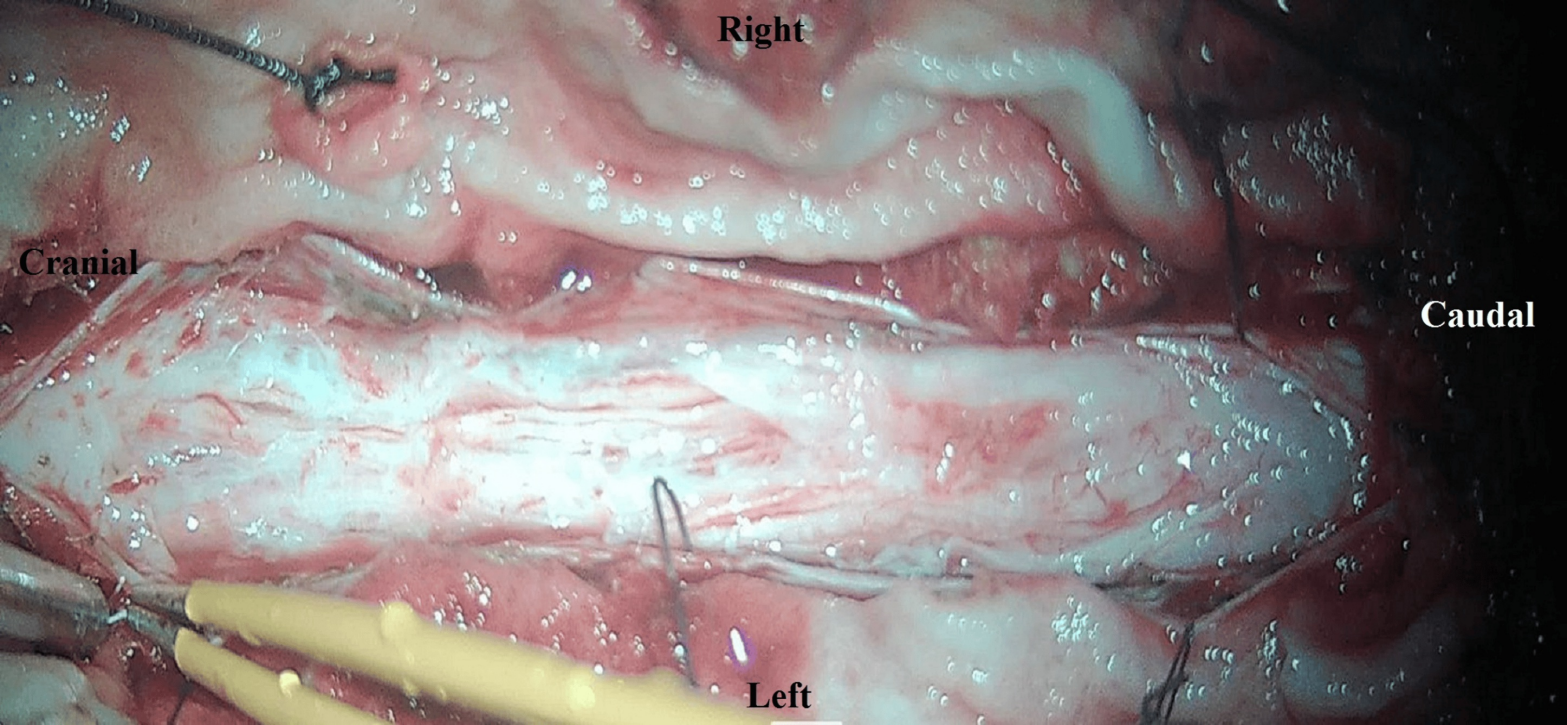
Intraoperative view of spinal adhesive arachnoiditis showing intradural, dense arachnoid adhesions encasing the nerve roots.

The patient gradually developed low back pain over the following years, which worsened with sitting and forward bending and was relieved by lying supine. She also experienced bladder sphincter dysfunction, difficulty walking, and progressive paraparesis. Neurological examination showed motor weakness, worse in the left lower limb than the right, and reflexes in both upper and lower limbs were normal. MRI of the lumbosacral spine showed an intradural lesion involving the cauda equina below the conus, extending from the inferior endplate of the L1 vertebral body to the superior endplate of L5. The lesion appeared heterogeneously hyperintense on T2 and STIR, isointense on T1, and showed intense heterogeneous contrast enhancement on post-gadolinium T1-weighted images (Figure 1). Whole spine MRI screening ruled out any dissemination. Based on these MRI findings, differential diagnoses of benign schwannoma, cauda equina myxopapillary ependymoma, and astrocytoma were considered. The patient underwent L2 to L4 laminectomy. Upon opening the dura, a significantly thickened arachnoid layer was observed. All roots of the cauda equina were thickened and densely enveloped by the arachnoid layer (Figure 2).

**Figure 3:**
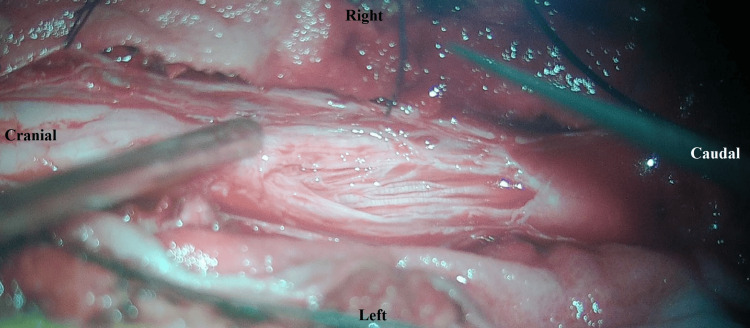
Adhesiolysis of encased nerve roots done.

**Figure 4:**
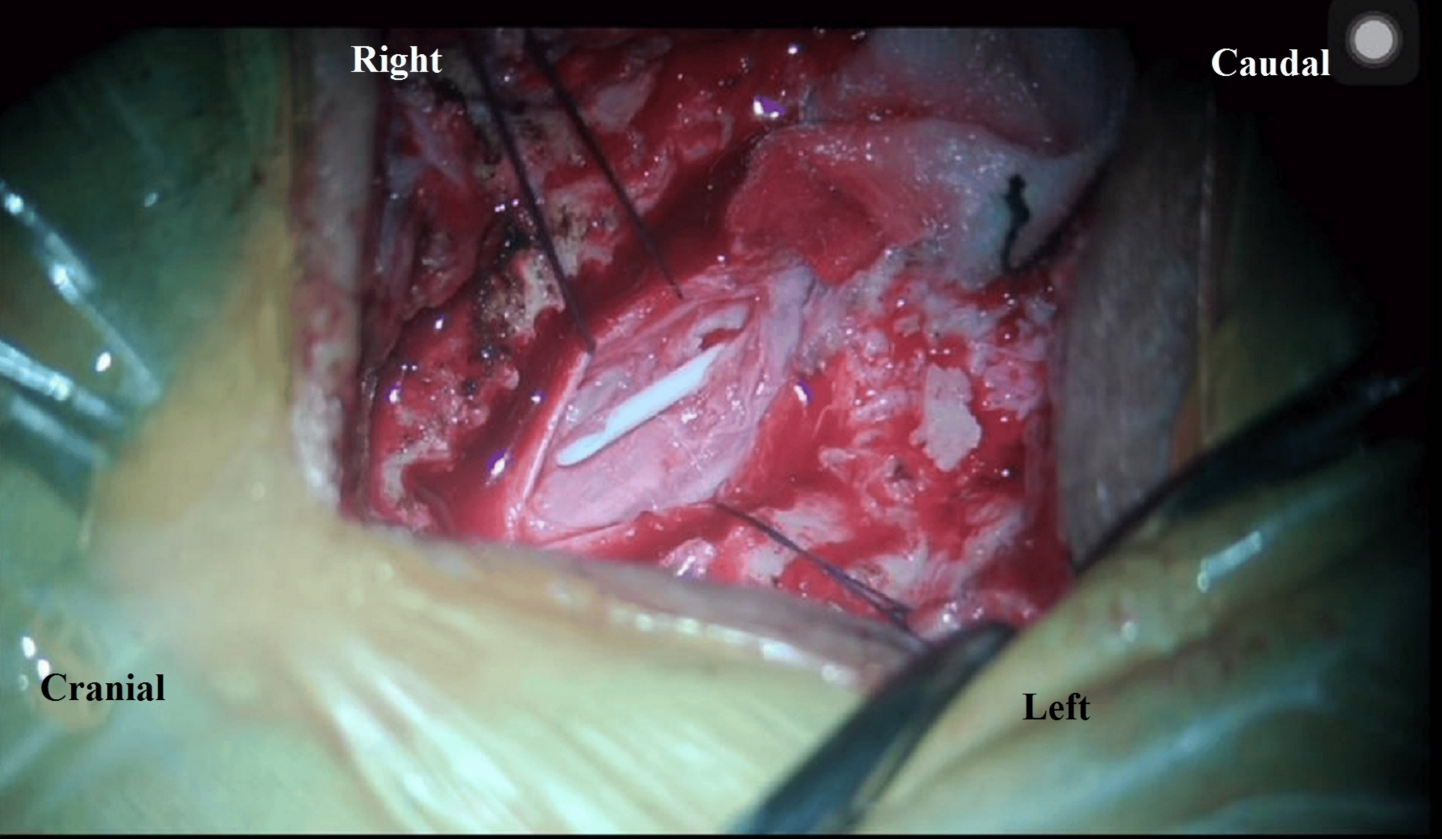
A retained proximal lumbar drain catheter was revealed after adhesiolysis of the encased nerve roots and was carefully removed.

**Figure 5:**
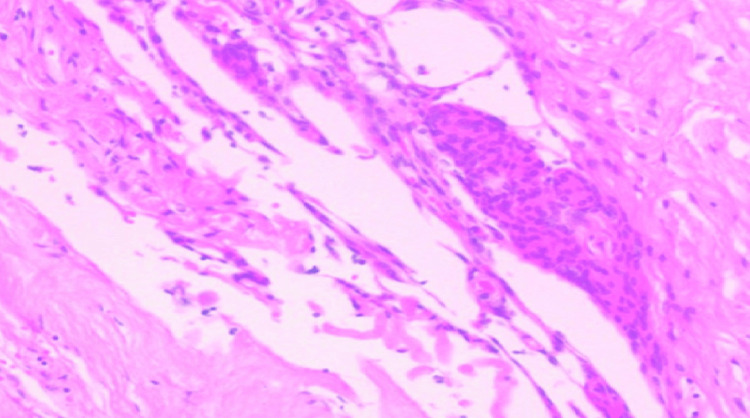
Hematoxylin and eosin staining of the arachnoid adhesions showing fibrous arachnoid thickening with inflammatory infiltrates.

Arachnoid adhesiolysis was performed safely (Figure 3), which, surprisingly, revealed a retained proximal lumbar drain catheter that was carefully removed (Figure 4; Video 1). There was no evidence of any intradural or extradural tumor. Biopsy taken from the thickened arachnoid for histopathological examination showed fibrous arachnoid tissue with scant inflammatory cells and no evidence of any malignancy or infective pathology (Figure 5). The postoperative course was uneventful, and the patient was discharged on oral medications. The patient showed gradual improvement in pain and lower limb power with physiotherapy and rehabilitation during regular follow-up for up to one year postoperatively.

**Video 1:** The retained lumbar drain catheter was removed after careful adhesiolysis of the encased nerve roots.

## Discussion

SAA is a rare, debilitating condition where the leptomeninges develop an inflammatory response to an irritant, causing intrathecal scarring that disrupts subarachnoid CSF flow and blood supply, leading to tethering and atrophy of neural tissue. The true incidence of this condition is significantly underestimated due to the missed diagnosis in subclinical cases or those in which the cause of paraparesis is thought to be spinal canal stenosis or space-occupying tumor [5]. The clinical presentation of SAA includes back pain, radicular burning pain in the distribution of the sciatic nerve, restriction of mobility in the lumbosacral spine, sensory-motor deficits in the radicular distribution, neurogenic bladder dysfunction, and pyramidal tract syndromes [4,5].

The pathogenesis of adhesive arachnoiditis occurs from an initial injury to the pia-arachnoid, initiating an immune response, causing arachnoiditis, characterized by a relatively oligocellular, fibrinous exudate, nerve root swelling with hyperemia, and development of radicular symptoms [5]. The lack of arachnoid vasculature results in fewer leucocytes, while circulating CSF dilutes phagocytes and fibrinolytic enzymes, which allows unregulated fibrin and collagen deposition and band formation. This process leads to adhesions of nerve roots to each other and/or to the dural sac, decreased blood supply, and tethering, encasement, and atrophy of the neural elements. There is a significant reduction in nutrient supply to the neural tissue due to the disruption of CSF flow [6].

The etiology of SAA includes infections (bacterial, tubercular, syphilitic), trauma (including iatrogenic), thecal contamination from spinal injection of lipoidal contrast agents, anesthetic drugs, or corticosteroids, and, rarely, spinal tumors [6-8], or even a retained foreign body, as in this case. Though SAA is rare, it is a recognized cause of neurological deficits following spinal anesthesia. It may result from direct trauma to neural tissue or, less commonly, from infection, epidural hematoma, cord hypoxia, or injection of anesthetic agents into or adjacent to a spinal canal with critical stenosis due to disc prolapse or osteophytes [9].

Rarely, SAA may occur as a result of aneurysmal subarachnoid hemorrhage, predominantly of the posterior circulation. It has been postulated that oxidative damage to neural elements caused by blood breakdown products can precipitate SAA [9], although conclusive evidence in its support is lacking.

Contamination of intrathecal or epidural local anesthetic solution with phenolic, acidic, and detergent contaminants has been hypothesized as an etiological factor [10]. Recently, alcoholic chlorhexidine cleansing agent has been implicated in causing severe SAA. Pooling of chlorhexidine on the skin should be avoided, allowing it to dry completely before any spinal procedure [11].

This case is unique and provides a new insight into the existing etiological factors for this rare condition. Foreign body reaction caused by a retained silicone lumbar drain catheter, causing chronic adhesive arachnoiditis, is an extremely rare finding. It also emphasizes the need for caution while inserting and removing a CSF drainage catheter of any size or material.

Contrast-enhanced MRI is the most important, non-invasive diagnostic modality [12]. Though its sensitivity and specificity in diagnosing SAA are reported as 92% and 100%, respectively, there is no consistent correlation of the extent of radiologic changes with patients’ clinical presentations [12]. MRI findings consistent with chronic adhesive arachnoiditis are grouped into three categories as proposed by Delamarter et al.: (1) peripherally adherent nerve roots to the thecal sac, giving an empty thecal sac appearance; (2) centrally adherent nerve roots within the CSF; and (3) thecal sac filled by matted nerve roots [12].

In this case, the hypointensity observed within the contrast-enhancing tissue on gadolinium-enhanced T1-weighted images most likely represents a retained lumbar drain catheter, as corroborated by the intraoperative findings. An X-ray or CT scan of the lumbar spine would have clearly shown the presence of the retained lumbar drain catheter. However, as there were no documented records from the previous surgery performed at a different center suggesting the possibility of a retained lumbar drain catheter, and the patient’s current symptoms and neurological assessment pointed toward a space-occupying intradural lesion in the lumbar spine, MRI of the lumbar spine with whole-spine screening was the only imaging study performed. This case thus highlights the importance of simple X-ray imaging, even when a spinal tumor is suspected, especially when the same spinal region has previously undergone a procedure such as temporary lumbar drain insertion.

Treatment of SAA is extremely difficult with limited options available at present. Patients with the most severe presentations often develop lasting disability despite aggressive treatment. Opioid analgesics, nonsteroidal anti-inflammatory drugs (NSAIDs), corticosteroids, and neural stimulation are among the most widely used treatment methods. Decompressive laminectomy and microsurgical adhesiolysis of the nerve roots and excision of space-occupying cysts, when present, are advocated, though outcomes remain generally poor. Many authors have reported modifications in surgical management with the aim of minimizing recurrence risk. Micro-adhesiolysis, Gore-Tex graft expansive duroplasty, and expansive laminoplasty, along with multiple tenting sutures of the Gore-Tex graft, have been proposed by Ohata et al. [13]. Mitsuyama et al. [14] have proposed a ventriculo-subarachnoid shunt for CSF diversion following microdissection of the thickened, adherent arachnoid. 

In this case, removal of the retained lumbar drain catheter prevented further irritation of the neural tissue and progression of adhesive arachnoiditis. Adequate safe adhesiolysis, along with postoperative physiotherapy and rehabilitation, gradually improved the patient's neurological symptoms to a certain extent.

## Conclusions

SAA is a pathology of various etiologies and neurological presentations that can lead to serious disability. Even though rare, it should be considered as a differential diagnosis in patients with progressively worsening pain and weakness of lower extremities, especially in ones with identifiable risk factors. MRI is crucial for establishing the diagnosis, allowing the identification of radiologic changes typical for SAA, which, at times, may mimic an intradural spinal tumor. X-ray or CT imaging, along with early clinical suspicion, often aids in identifying a retained foreign body that could be the irritant agent causing arachnoiditis in a case where a clinical history of spinal instrumentation is present, thus alerting the surgeon to a different, rather benign diagnosis.

Despite the current knowledge of the nature of adhesive arachnoiditis, there are still no established guidelines on the prophylaxis or treatment of this rare and unpredictable disease. While taking informed consent for any spinal anesthesia or lumbar CSF drainage procedures, patients should be informed about the possibility of delayed, even permanent, neurological deficit.
